# The future of fungal diagnostics: What’s next in laboratory testing for invasive fungal infection?

**DOI:** 10.1093/mmy/myag085

**Published:** 2026-08-22

**Authors:** Rachel Southern-Thomas, Simran Goyal, Anna Louise Wild, Neil Robert Hope Stone

**Affiliations:** Division of Infection, University College London Hospitals, London, NW1 2PG, UK; Division of Infection, University College London Hospitals, London, NW1 2PG, UK; Department of Clinical Microbiology, Queens Medical Centre, Nottingham, Nottinghamshire, NG7 2UH, UK; Division of Infection, University College London Hospitals, London, NW1 2PG, UK; Centre for Clinical Microbiology, University College London, London. NW3 2PF, UK

**Keywords:** invasive fungal infections, molecular diagnostic techniques, rapid diagnostic tests, antifungal agents, microbiological techniques, fungal diagnostics

## Abstract

Invasive fungal infection (IFI) is responsible for millions of deaths globally each year. Prompt diagnosis of IFI is critical, with delay associated with increased mortality. Traditional, culture-based diagnostics are relatively slow and insensitive. Novel molecular techniques, biomarkers and point-of-care tests hope to overcome these limitations. However, these newer methods come with issues of cost, accessibility, and standardisation. This review briefly lays out the current diagnostic landscape, highlights important changes to standards of care, then explores new developments and the future of fungal laboratory diagnosis.

## Introduction

Prompt and accurate diagnosis of invasive fungal infection (IFI) is critical. Delays to treatment are associated with higher mortality, particularly in immunocompromised patients.^1^ Accurate diagnostics enable antifungal stewardship by cessation or de-escalation of antifungal therapy. The challenge of identifying and treating IFI has never been more salient: the pool of susceptible hosts is widening due to increasing iatrogenic immunosuppression, comorbid populations and the ongoing HIV pandemic. Compounding this, climate change is altering fungal ecological niches, challenging accepted wisdom around limited geographical distribution of fungal threats.^2–4^ This is typified by the emergence of previously unknown species, such as *Emergomyces*, on four continents,^5^ as well as the dramatic global spread of the multidrug-resistant yeast *Candidozyma auris*. ^6^

Therapeutic options for IFI are generally limited to three major drug classes (azoles, echinocandins, and polyenes). Resistance to, or intolerance of, any one of these narrows already limited treatment options, with pan-resistant fungal infections now a clinical reality.^7^ Factors contributing to the rise in antifungal resistance include widespread use of antifungals in the agricultural and animal industries; use of antifungal prophylaxis; development of hypermutating strains and empirical treatment of at-risk populations, alongside the emergence of intrinsically drug resistant species.^2,8^ Judicious use of antifungals is essential: both to prevent further selective pressure driving drug resistance and to provide effective, individualised treatment.

No single, readily available test can reliably diagnose cases of IFI, so a multi-pronged approach is essential. This is especially true of mould infections such as aspergillosis which, unlike candidiasis, generally do not yield a positive blood culture. The consensus framework classifying the likelihood of invasive fungal disease using ‘proven’, ‘probable’ and ‘possible’ criteria in the 2020 updated European Organization for Research and Treatment of Cancer and Mycoses Study Group (EORTC/MSG) guidelines has become a standard of care and widely adopted in clinical practice worldwide.^9^ Alongside mycological diagnosis (histopathology, culture, or other tests), clinical features and host risk factors are considered in the probability of the presence of a severe fungal infection. This is a useful framework for both research and clinical settings but is limited by the lack of highly sensitive and specific diagnostics available.

However, such diagnostic frameworks continue to adapt with the advent of newer, mainly molecular, diagnostics for IFI. The recent British Society for Medical Mycology (BSMM) best practice recommendations advised on how to position serological and molecular methods to improve fungal diagnosis, in conjunction with culture-based techniques.^10^ Furthermore, in 2025, WHO released a landscape report on the commercially available diagnostic tests for the Fungal Priority Pathogens list which acknowledged the importance of culture-based diagnosis, whilst encouraging use of novel tests.^11^

Several strategies for antifungal treatment in the immunocompromised have been developed aiming to position existing diagnostics in order to maximise clinical impact. Prophylaxis is widely utilised, particularly in patients undergoing treatments for acute leukaemia or haematopoietic stem cell transplant.^12^ However, drawbacks include drug toxicities, cost (directly to patient and/or health system depending on the setting), drug–drug interactions and breakthrough infections. Surveillance with pre-emptive therapy is an alternative strategy to universal prophylaxis, involving regular biomarker surveillance in patients at high risk for IFI, with a positive result prompting investigation and possibly treatment. This is currently being investigated in a phase III randomised trial in acute myeloid leukaemia patients compared to a prophylaxis arm.^13^ An empirical therapy approach, with fever not responsive to antibacterials in high-risk patients treated with empiric antifungals, is widely used. Limitations include resultant overuse of antifungal drugs with their associated toxicities and consequent suboptimal antifungal stewardship.​^14^ And finally, the ideal, but often hard to achieve, goal of clinical mycology is a targeted therapy strategy, whereby antifungals are directed against a proven fungal pathogen. Currently, limitations of diagnostics to definitively prove IFI in a timely manner prohibit use of this as a sole policy in high-risk cohorts, where delayed treatment initiation can prove fatal.

Given these limitations, it is critical to use available diagnostics in the most effective way, avoiding missed diagnoses in high-risk patients while safeguarding antifungal stewardship. This review focuses on laboratory diagnostics for IFI currently available for clinical use or in development, including biomarkers, molecular methods and microfluidics, as well as looking to the future of the field with the latest developments in novel diagnostic techniques including volatile organic compounds (VOCs) and fungus-reactive T cell based assays.

## Current landscape: Culture, identification, and susceptibility testing

**Figure 1:**
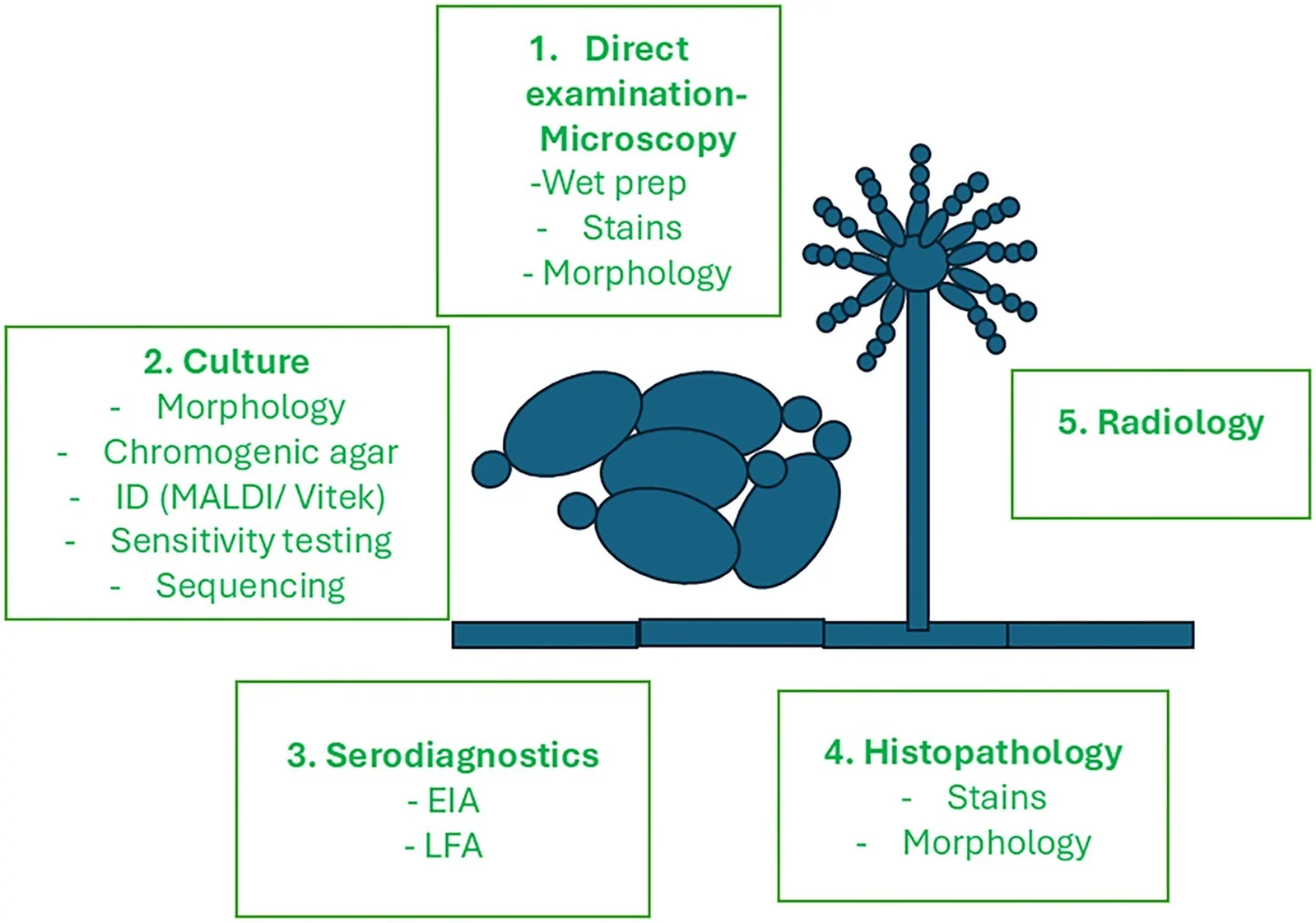
Summary of conventional mycological tests available for fungal diagnosis. EIA = Enzyme immunoassay; LFA = lateral flow assay. This is an image of 5 broad categories of fungal diagnostics including microscopy, culture, serology, histopathology and radiology

Culture, from blood or invasive sampling, remains the backbone of clinical mycology. This allows for species identification and antifungal susceptibility testing, which is still dependent on culture for the majority of pathogens. However, positive yield is poor, especially in the case of invasive mould infection.^15^ Furthermore, invasive sampling may be dangerous or contraindicated in immunosuppressed patients, who are often at risk of complications, in particular, bleeding. Figure 1 summarises the conventional mycological tests available in most laboratories.

Historically, once culture of an organism was obtained, identification was made using macro- and microscopic morphology^9,16^ or biochemical reaction profiles.^17^ These techniques have largely been superseded by mass spectrometry (MS)-based identification.

## Matrix-assisted laser desorption ionization time of flight MS

Matrix-assisted laser desorption ionization time of flight (MALDI-TOF) MS is widely used for fungal species identification by analysing conserved ribosomal proteins to generate species-specific spectral profiles based on the mass-to-charge ratio of laser-ionised particles.

Two MALDI TOF platforms, Biotyper (Bruker, Billerica, MA) and Vitek (BioMérieux, Marcy-L’Etoile, France) dominate the market for MALDI-TOF MS for diagnostic microbiology laboratories and both perform well for yeasts.^18^ They are increasingly able to reliably identify moulds with recently updated databases.^19^ MALDI-TOF MS is being explored for determining antifungal susceptibilities with short pre-incubation times (3–6 h) by detecting changes in spectral profiles of isolates exposed to antifungals.^20^ However, further development is needed to expand its real-world applicability.^21^ The strengths and limitations of MALDI-TOF and other existing mycological tests are outlined in Table 1.

**Table 1:** Summary of conventional mycological testing for invasive fungal infection.

Method	Description	Pros	Cons
**Culture**	Sterile sites including blood, CSF, joint fluid, can be cultured as well as urine, BALF, tissue samples	Specific Species identification Low cost Allows for susceptibility testing	Low sensitivity Relatively long turnaround time
**Direct microscopy and histopathology**	Microscopic examination of blood or sterile site tissue samples for fungal elements. Including special staining e.g., Calcofluor white	Low cost Rapid	Limited sensitivity if fungal burden is low High levels of mycology expertise needed Invasive sampling (for tissue biopsy)
**MALDI-TOF MS**	Mass Spectrometry following positive blood cultures can be used to identify fungal pathogens	Rapid (<10 min) Specific, with ability to differentiate phenotypically similar species Growing potential for susceptibility testing	Reliance on positive culture. Variability based on growth medium (moulds) Limited to known fungal sequences Small mould databases resulting in risk of misidentification High initial access cost
**BDG**	Enzyme immunoassay detecting the polysaccharide cell wall component present in many fungi	Easily obtainable sample High NPV	Low specificity Lower specificity in certain *Candida* species such as *Candida parapsilosis, C. auris*^210,211^ Unsuitable to detect *Mucorales, Blastomyces* spp. and *Cryptococcus* spp.
**Galactomannan**	Enzyme immunoassay detecting this fungal antigen, often used in serum and BALF	Easily obtainable sample High NPV in BALF Can be used on many sample types	Limited sensitivity and specificity Impacted by concomitant mould active antifungals Limited range of species detected, e.g., *Aspergillus* spp, *Fusarium* spp

MALDI-TOF MS = Matrix-Assisted Laser Desorption/Ionisation Time of Flight Mass Spectrometry, BDG = 1,3-Beta-D-glucan (BDG) assay, NPV = negative predictive value, BALF = Bronchoalveolar Lavage Fluid.

## Serodiagnostics: Antibody and antigen-based tests, including lateral flow assays and biomarkers

Serological testing can provide valuable information without the need for invasive sampling. The 1,3-Beta-D-glucan (BDG) assay e.g., Fungitell (Associates of Cape Cod, MT) a colorimetric assay, and Wako (Fujifilm) using a turbidimetric method, detects this polysaccharide cell wall component present in many fungi, with *Cryptococcus* spp. and *Mucorales* the notable exceptions. Galactomannan (GM) is a polysaccharide found in many fungal cell walls, with some important exclusions such as the *Mucorales* order. The Platelia assay, which uses EBA-2 monoclonal antibody (mAb) to detect GM antigen, has been widely adopted for GM detection in bronchoalveolar lavage fluid (BALF) and serum but may have reduced sensitivity in patients on antifungal therapy.^22^

Detection of antibodies against *Aspergillus* spp. forms the key laboratory component of diagnosis for chronic forms of aspergillosis.^23,24^ Manual precipitin assays have now been superseded by automated serological tests, namely enzyme immunoassays (EIAs).^25^ There is signal that *Aspergillus* IgG could be used adjunctively in non-neutropenic invasive aspergillosis, either as a lateral flow assay (LFA)^26^ or the ThermoFisher Scientific ImmunoCAP.^27^ However, utility remains limited, like other serological tests, by host immune system activity, with low overall seroprevalence even in non-neutropenia. Another issue is that most assays are directed against *Aspergillus fumigatus*, with lower sensitivity for identification of non-fumigatus spp.^28^

For diagnosis of invasive candidiasis*, the Candida albicans* germ tube-specific antibody has an overall sensitivity of 66% and specificity of 76%.^29^ It has the advantage of being indifferent to use of antifungal agents or *Candida* colonization, however a clear limitation is that it only detects a single species of *Candida*. ^30^

Another *Candida* antibody test is the anti-mannan IgG test, often used in conjunction with the mannan antigen (polysaccharide target of the *Candida* cell wall) test for a combined sensitivity and specificity of 83% and 86% respectively for candidaemia.^31^

With regards to dimorphic fungi, EIA on urine or serum is often used in the initial screening for diagnosis of *Histoplasma* and *Blastomyces* given their high sensitivity (79%–82% and 56%–93%) and non-invasive nature. A limitation is cross-reactivity across these two species. Older methods for antibody detection, such as immunodiffusion and complement fixation are often used as confirmatory or adjunctive tests. ^32,33^ Regarding *Coccidioides*, serology remains the most trusted method of detection.^34^

Host biomarkers, such as cytokines and chemokines, show potential for fungal diagnosis, with studies demonstrating that the pattern of interleukins is able to distinguish invasive from allergic bronchopulmonary aspergillosis. Studies have now also shown that host cytokine and chemokine profiling can help differentiate between fungal and bacterial bloodstream infections.^35,36^ However, at present, use remains limited to research settings.

LFAs are rapid, affordable point-of-care tests, particularly useful in low-resource settings, where access to molecular methods may be limited. The most widely adopted LFA is the Cryptococcal Antigen (CrAg) test (IMMY). Evidence for its accuracy in serum and CSF is well established, both in HIV^37^ and non-HIV related cryptococcal disease.^38^ There is also growing evidence to support the use of whole finger-prick blood in HIV-negative patients, which could decrease costs further.^39^

There are also commercially available LFAs detecting *Aspergillus* GM antigens that have been validated in serum and BALF: Sõna *Aspergillus* GM LFA (IMMY); Fungadia *Aspergillus* antigen (Gadia); AspLFD (OLM Diagnostics), clarus *Aspergillus* Galactomannan Enzyme Immunoassay (IMMY); and QuicGM™ *Aspergillus* GM LFA (Dynamiker).^40–42^ Combining an *Aspergillus* GM LFA with GM EIA shows potential as a rule-out test for invasive aspergillosis.^43^

LFAs have been developed for some endemic mycoses. The sōna *Coccidioides* Antibody LFA Sōna (IMMY) is a research use only test for rapid *Coccidioides* antibody detection.^44–46^ Two CE-marked *Histoplasma* LFAs are available: MVista® *Histoplasma* urine antigen lateral flow immunoassay (MiraVista Diagnostics) and OIDx H. capsulatum antigen qualitative LFA (Optimum Imaging Diagnostics)^47,48^ These *Histoplasma* LFAs have reduced sensitivity in immunocompetent patients.

There are LFA in development against *Mucorales* spp., ^49,50^*C. albicans* enolase Ab, ^51^ and anti-P Ab in *Pneumocystis jirovecii* pneumonia (PJP).^52^ although all are in early stages and clinical utility in a real-world setting remains to be seen.

## Novel mAb assays

Researchers have investigated other mAbs for improved detection of novel antigens. For example, an LFA utilising JF5 (AspLFD; OLM Diagnostics) has a high specificity and low cross-reactivity with non-*Aspergillus* fungi, though further studies are needed to prove clinical benefit.^53^ More recently, this antibody has also been developed into an EIA (Euroimmun Medizinische Labordiagnostika AG), which has shown comparable performance to GM in both serum and BALF.^54–56^ Other mAbs, such as AP3, have been shown to have specific affinity for GM in certain *Aspergillus* species, aiding in more specific diagnostics.^57^

**Figure 2:**
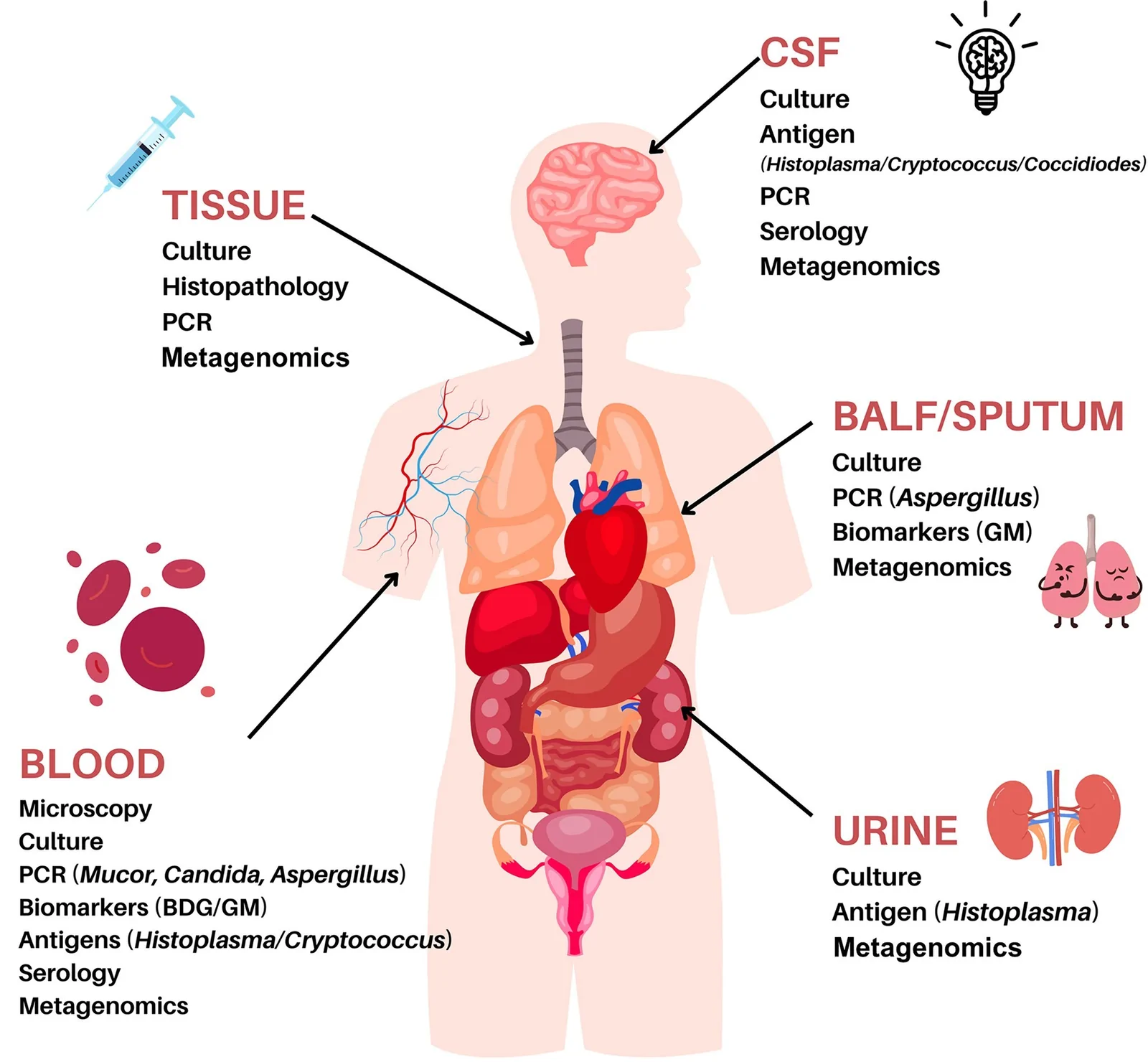
Diagnostics for invasive fungal infection by sample type. CSF = cerebrospinal fluid; BDG = 1,3-Beta-D-glucan; GM= galactomannan. This is a graphic image of human sample types used for fungal diagnostics - blood, BAL/sputum, urin, CSF and tissue

Figure 2 shows the diagnostics for IFI split by sample type. Urine is readily obtainable and non-invasive, however the Platelia GM assay does not perform well on this sample type, even after accounting for creatinine level.^58^ Galactofuranose contributes to the structure of GM in a variety of pathogenic fungi and is not able to be synthesised by mammalian hosts, so has been a target for antibody development.^59^ An LFA to detect galactofuranose in urine (MycoMEIA, Pearl Diagnostics, Baltimore MD) has demonstrated sensitivity and specificity for diagnosis of *Aspergillus* lung infection in a cohort of 78 patients with suspected IFI, and has now been Federal Drug Administration (FDA) approved.^60^ However, further studies are required to demonstrate real-world, clinical utility.

Other extracellular polysaccharides (EPS) antigens have been targeted during hyphal growth phase of some moulds for fungal diagnosis.^50^ A lateral flow device has recently been developed using TG11, a mAb that binds to specific EPS antigens secreted during *Mucorales* hyphal growth phase. This can be used in both serum and BALF for rapid mould detection, although at the time of writing was not commercially available and efficacy is yet to be demonstrated in the clinical setting.^49^

A workflow using sequential LFAs with mAb technology has been able to quickly differentiate genus of fungi within the *Mucorales* order. If applied clinically, this could enable rapid targeting of antifungal therapy.^61^

## Molecular methods

Nucleic-acid-based detection methods are advancing rapidly. Polymerase chain reaction (PCR) assays target known sequences, whereas next generation sequencing (NGS) methods can produce unbiased sequences and longer reads. Quantitative PCR (qPCR) can be used for disease monitoring and distinguishing colonisation from infection. PCR assays may be directed toward one or multiple (multiplex) genus or species of concern or can be ‘panfungal’. Development has focused on *Candida* and *Aspergillus* spp. as the pathogens causing the highest disease burden.^62^

DNA-based methods are dependent on the quantity and method of DNA extraction from the clinical sample, with reduced DNA yield from formalin fixed samples.^63^

PCR is now an indispensable part of the fungal diagnostic algorithm in resource-rich countries. The commercially available PCRs and their positioning for various IFIs have been explored in recent reviews,^64–66^ and the BSMM guideline has a separate appendix focusing on prioritisation of testing in lower-income settings.^10^ The most important PCR panels are detailed briefly here, before moving on to PCR innovations.

## PCR-based diagnostics—*Candida, Aspergillus*, PJP, and *Mucorales*

*Candida* PCR has now been adopted in many laboratories and is recommended by the BSMM.^10^ There are a number of commercial and research-stage panels enabling speciation of yeasts from serum or following positive blood culture.^67^

T2Candida® uses T2 Magnetic Resonance (T2MR) technology combined with PCR. Whole blood samples are analysed to detect the presence of five common *Candida* species (*C. albicans, C. tropicalis, C. parapsilosis, C. krusei, and C. glabrata*).^68,69^ The test requires 2–4 ml of blood, delivering results within 5 h, with a detection limit as low as 1 CFU/Ml.^70,71^ A multi-centre trial for T2Candida reported overall sensitivity of 91.1%, specificity of 98.1% on *Candida*-spiked blood culture samples.^72^ The fast turnaround time (TAT) and high specificity of this tool allows for cessation of antifungals in negative cases.^73^ A further advancement has been the development of the new T2Cauris™ *Candidozyma auris* (*C. auris*) panel​​ which has FDA Breakthrough Device designation but is not yet licensed for clinical use. ^74^

Other technologies harnessing magnetism include the M1-PCR, which has outperformed qPCR in terms of sensitivity. ^75,76^ Benefits of magnetism-based PCRs include quick TAT (3.5 h for M1-PCR) and high sensitivity. However, they are non-quantitative techniques and require fresh preparation of the magnetic beads.

There is limited data validation for other commercial *Candida* PCR assays.^77^ The CandID from OLM/IMMY can detect *C. albicans, C. glabrata, C. parapsilosis, C. tropicalis, C. dubliniensis*, and *C. krusei* from serum samples, with a recent study reporting 100% sensitivity for detection of proven candidiasis and 83.3% for proven/probable, when tested in a selected cohort of BDG-positive intensive care patients.^78^ Similarly, the Bruker Fungiplex® *Candida* assay demonstrated a sensitivity of 100% and specificity of 94% (*n* = 58) compared to blood cultures in intensive care patients.^79^

Others are streamlining PCR: a direct-to-PCR proprietary buffer has been developed to enable extraction-free PCR. This lyses microbial walls while leaving DNA intact, allowing amplification to be carried out without prior purification steps. In a small study of spiked and clinical samples, this method was able to detect clinically relevant *Candida* spp. with similar diagnostic accuracy to the Qiagen platform.^80^

*Candida auris* has spread globally and there has been a contemporaneous explosion of diagnostic tools investigated in response.^81^ PCR screening and surveillance is now commonly performed for *C. auris*, with cutaneous swab and urine samples validated in diverse cohorts.^82,83^ There is a wide variety in sensitivity and specificity between current PCR assays, both commercially available and in-house.^84^ However, performance overall appears impressive, with a 2022 meta-analysis showing pooled sensitivity and specificity for PCR of 94% and 99%, respectively.^85^ This trend has been maintained over time, with similar results in a 2025 literature analysis.^86^

In particular, qPCR for *C. auris* is desirable to rapidly detect colonised patients in an outbreak scenario or for screening.^87–89^ However, qPCR is often complex, requiring multiple channels and prolonging TAT. A 2025 study described design of a multiplex qPCR which used melting curve analysis to detect five *Candida* species in a single channel, on a single run.^90^ This approach requires further validation in clinical samples but would likely be more cost-effective than magnetic resonance PCRs and would not require additional lab equipment.

PCR of respiratory samples is a well-established tool in the diagnosis of PJP. There are now an array of commercially available options with CE-IVD (in vitro diagnostic) marking, and one FDA-IVD accredited multiplex PCR panel which includes PJP, summarised in a recent minireview of PJP diagnostics.^91^ Studies have found that PCR outperforms immunofluorescent staining in terms of sensitivity.^92,93^ qPCR is preferred, as the cycle threshold can be used to help differentiate between respiratory tract colonisation and infection.^94,95^ Furthermore, investigators have suggested that normalising fungal load by cell could improve performance of these cutoffs.^96^

*Aspergillus* PCR is included as a mycological criterion in the latest EORTC/MSG guidelines ^9^ and in the BSMM best practice recommendations.^10^*Aspergillus* PCR in blood and BALF can be used to rule -in infection in cases of high pre-test probability. Conversely, where lower suspicion exists, testing has a good negative predictive value, allowing antifungal cessation. Standardised methodology has been introduced, and commercial assays are now widely available, however caution must be taken in interpretation. A longitudinal external quality assessment of centres using *Aspergillus* PCR noted that new centres had higher false positive rates for in house qPCR, and that detection of non-fumigatus species was inferior to that previously reported.^97^

Mucormycosis is a devastating fungal infection caused by species of the order *Mucorales* that affects immunocompromised hosts.^98^ There is no current biomarker for *Mucorales* spp., making PCR an attractive target,^99^ but efforts for standardization lag behind those of *Aspergillus*.^100^ Various commercially available assays have been studied, including MucorGenius®, which showed good sensitivity and specificity in BALF samples of immunocompromised patients^101^ However, it underperformed compared to in-house qPCR in another study.^102^

Due perhaps to the overlap in host risk factors, companies and laboratories have combined *Mucorales* and *Aspergillus* spp. detection into one PCR workflow. Examples include the CE-IVD MycoGENIE® *Aspergillus* species*-Mucorales* species (ADEMTECH SA), which performed well in comparison to in-house PCR.^103^ Other similar kits such as the Fungal Plus PCR profile (Eurofins Viracor Inc) are commercially available.^104^*Authors* using an in-house *Aspergillus* and *Mucorales* qPCR on blood samples noted an association between increased fungal load and subsequent mortality. If borne out in further studies, this could allow identification of the highest risk patients with tailored management strategies.^105^

A 2025 meta-analysis demonstrated pooled sensitivity and specificity of 51% and 97% for blood PCR in diagnosis of mucormycosis, with a sensitivity increment of 14% with every additional blood sample tested and higher sensitivity still for BALF samples.^106^

Twice-weekly Mucor qPCR has been trialled as a screening strategy for high-risk patients in France; they found reduced mortality in their cohort compared to the national data registry cohort, but further studies are needed to replicate this result.^107^

In the last year, four qPCR assays have come to market (Eurofins Viracor) for detecting and differentiating 3 major dimorphic fungi (*Histoplasma, Blastomyces*, and *Coccidioides)* and well as speciating *Cryptococcus* (differentiating between *C. gattii* and *C. neoformans*).^108^ Suitable sample types are BALF, sputum, or CSF. This panel of assays is not currently routinely available but offers promise with regards to timely diagnostics and subsequent antifungal stewardship.

## Detection of antifungal resistance using PCR

To decide on best antifungal therapy, it is paramount to not only ascertain a rapid diagnosis at species level, but also to understand the likely resistances. Historically a blind spot of molecular technologies, research is beginning to close this key gap between molecular diagnostics and conventional mycological tests. Real-time PCR kits such as AsperGenius Resistance TR multiplex (PathoNostics) and Fungiplex *Aspergillus* Azole-R (Bruker) are able to detect specific resistance genes including cyp51A for azole resistance. ^109,110^ Other species have been studied, including a promising PCR for a gene linked with fluconazole-resistant *C. parapsilosis*^111,112^; and CDR1 and CDR2 expression also linked to *C. albicans*.^113^

While detection of isolated mutations by PCR is useful, our understanding of fungal antimicrobial resistance mechanisms remains incomplete, and formal susceptibility testing from culture remains necessary in most cases. In the future, advanced sequencing and accurate assembly may be able to identify and categorise relevant mutations and translocations.^114^ Were resistance mechanisms to be reliably detected the same day a sample is received, targeted antifungal treatment could be given, not only impacting morbidity and mortality, but also improving antifungal stewardship.

## Broad multiplex and panfungal PCRs

Combining multiple PCRs into one assay is a common technique for improving laboratory workflows. Many groups have created multiplex assays which focus on *Candida* and *Aspergillus* as the most clinically problematic genera. A quadruplex assay to detect two *Candida* and two *Aspergillus* species simultaneously in spiked whole blood has demonstrated proof of concept, with plans to convert this to real time PCR. ^115^ Combining LFAs with PCR steps has been used to identify *Candida* and *Aspergillus*. Authors developed a local pathway in which they first performed *Candida* and *Aspergillus* multiplex PCR followed by repeated LFAs and targeted PCR to rapidly detect these fungi at species level. One issue common to many PCRs is the difficulty of separating or disregarding the human DNA in samples. This led a low sensitivity of 47.8% in this study, but identification was highly specific 99.4%, displaying potential as a rule-in test.^116^

Panfungal assays target highly conserved DNA regions, with 18S ribosomal RNA and associated Internal Transcribed Spacer regions the best described.^117^ This method should only be applied to sterile samples, as detection of commensal or environmental organisms risks unnecessary use of antifungals.^118^ The EORTC advises using panfungal assays only when fungal elements are seen on microscopic examination,^9^ as low yield of panfungal PCR in formalin-fixed paraffin embedded samples has been noted when fungal elements are not seen on microscopy.^119^

## Recent innovations and future directions

### Cell-free DNA PCR

It is possible to detect free fungal DNA fragments, or cell-free DNA (cfDNA), in clinical samples, such as plasma, by employing targeted PCR or untargeted NGS methods. cfDNA is already widely used in oncology and obstetrics for detection of chromosomal abnormalities and shows promise in the infection field.^120–122^

While not yet available for widespread clinical use, studies have demonstrated proof of concept and utility. A large single centre study of 438 patients testing in-house fungal cfDNA PCR panel using plasma demonstrated 88.5% concordance with invasive sampling. Authors noted a trend towards greater discordance in non-immunocompromised hosts. They suggest that invasive collection could be avoided in immunocompromised hosts with positive cell free DNA mould panel, excepting cases of suspected fungal sinusitis or limb infection.^123^

Authors from the same institution have recently reported on a *Mucorales* cfDNA PCR assay which remained accurate even in patients on mould-active prophylaxis. Similar to previously, sensitivity was reduced in immunocompetent patients, and those with isolated pulmonary disease.^124^

An in-house multiplex panel was developed for use on plasma and was highly specific (99.5%) for IFI, with sensitivity lower (56.5%), partially explained by insufficient plasma volume. The authors went on to demonstrate clinical feasibility and utility of their assay in suspected IFI, with a positive change in management in 42.6% of those testing positive for cfDNA ^125^

Others have developed workflows for cfDNA PCR that have enabled accurate detection of *Aspergillus*, outperforming GM with a sensitivity of 86% and specificity of 93%^120^

CfDNA PCR on plasma showed improved specificity in PJP diagnosis compared to BDG testing, but low sensitivity when probable cases were taken into account.^126^ Sampling via bronchoscopy remains the most important diagnostic tool for PJP, but if able to demonstrate improved performance, plasma cfDNA could be used in cases where this is contraindicated.

While most studies focus on adults, a similar fungal cfDNA multiplex assay has also been applied to a paediatric oncology population with encouraging results.^127^

Benefits of plasma cfDNA include avoidance of invasive sampling, lower risk to patient, lower cost and potential for quick TAT. There is hope that fungal cfDNA could be serially quantified, both to predict diagnosis and as a marker of disease activity over time, though this is not yet clinically validated.^128,129^

Clinical use is hindered by challenges common to most molecular techniques such as low fungal DNA abundance and contamination from human DNA, environmental and commensal microorganisms. Advances in bioinformatic workflows have been improving interpretation of cfDNA results in other medical fields and could help to overcome some of these limitations.^130,131^ Strategies to overcome the barriers to clinical adoption include improving pre-analytical conditions, optimising DNA preparation methods, and standardising workflows across laboratories.^121^

### Metagenomic next-generation sequencing

Metagenomic Next-Generation Sequencing may be targeted (tNGS) or untargeted (mNGS). In tNGS, or amplicon-based NGS, ITS or 18S ‘barcodes’ can be used to identify fungal reads which are subsequently sequenced. Conversely, the untargeted ‘shotgun’ approach identifies all genetic material in a sample and is therefore agnostic for any potential pathogens. This allows for rapid, broad-spectrum pathogen detection for complex cases with diagnostic uncertainty. Third-generation sequencing is the latest iteration of NGS, allowing for faster sequencing and longer reads. Key benefits of metagenomics include: no need for a culture step, option of non-invasive testing, ability to identify co-infections and suitability for diagnosing rare or unexpected pathogens, especially in immunocompromised patients.^132^ Commercially available platforms which allow metagenomic sequencing are increasing in number and include Oxford Nanopore Technologies, DISQVER® (cfDNA, Noscendo GmbH), iDTECT® Dx Blood (PathoQuest, used for quality control rather than clinically), and Karius Spectrum™ (Karius® cfDNA).

A study comparing mNGS to traditional culture methods for diagnosing infections in immunocompromised patients demonstrated a higher sensitivity of mNGS across all sample types (88.0% vs 26.3%). mNGS positively impacted patient management in 76.3% of cases, with treatment adjustments based on mNGS results, including antibiotic de-escalation in nearly 20% of patients, demonstrating its role in targeted, effective care.^133^

The metagenomic approach can be used to characterise typical pathogens in BALF samples of suspected respiratory infections and has greater sensitivity than culture.^134^

2022 meta-analysis focusing on BALF demonstrated a pooled sensitivity of 78% and specificity of 77%. The detection rate was increased in immunocompromised patients.^135^

tNGS performs well compared to traditional methods for diagnosing fungal lower respiratory tract infections, with a much reduced TAT (7 h from 2*–*7 days), however caution must be exercised with pickup of colonising organisms.^136^ Table 2 summarises this and other recent studies utilising metagenomics on BALF or CSF for diagnosis of IFI.

**Table 2:** summary of recent studies published utilising metagenomic sequencing for fungal diagnosis on bronchoalveolar lavage fluid (BALF) and cerebrospinal fluid (CSF) and their reported sensitivities and specificities.

Year published and study country	Study design	Specimen type and sequencing method	Sensitivity	Specificity	Comments and reference
2024, China	Retrospective analysis. ICU, non-neutropenic Chronic Obstructive Pulmonary Disease patients. 62 cases of IPA and 64 cases of non-IPA.	BALF, mNGS	70.9%	71.8%	Surpassed CMTs for diagnosis of IPA.^212^
2024, China	Retrospective analysis. *n* = 140 allogeneic Hematopoietic Stem Cell transplant recipients	BALF, mNGS	85.3%	98.1%	Improved sensitivity compared to CMTs. Lower sensitivity of 54.5% was noted for *Aspergillus* on mNGS.^213^
2025, China	Multicentre *n* = 253 patients with suspected pulmonary fungal infection	BALF mTGS	78.1%	90.5%	Shortened TAT compared to CMTs.^136^
2025, China	*n* = 84 patients with pulmonary cryptococcosis and *n* = 84 controls with other pulmonary diagnoses.	BALF, mNGS	85.7%	100%	Higher sensitivity noted in patients with symptoms or multiple radiological pulmonary lesions.^214^
2025, China	Two centre, non-HIV *n* = 168 propensity matched with and without PJP	BALF, mNGS	100%	96.4%	Outperformed BDG and Grocott staining of BALF for diagnosis of PJP.^215^
2025, China	Prospective, observational study *n* = 118 lung cancer patients with suspected PJP	BALF, mTGS	100%	100%	Excluded patients who had received PJP-active antimicrobials prior to sampling. ^216^
2025, China	Prospective, single centre. *n* = 327 non-neutropenic patients with suspected IPA	BALF, mNGS	80.6%	59.0%	Improved sensitivity and lower specificity of mNGS compared to combined CMTs which showed 61.9% sensitivity and 83.0% specificity. ^217^
2025, China	Retrospective case series of 115 patient with suspected pulmonary fungal infection	BALF, mNGS, tngs	95.1% (mNGS) 95.08% (tNGS)	90.7% (mNGS 85.2% (tNGS)	Detected mixed infections successfully.^218^
2025, China	Retrospective analysis. 65 patients with suspected CNS infection	CSF, mNGS	96.7%	100%	Lower sensitivity than the highly sensitive CrAg test.^219^

CMT = Conventional Mycological Tests, IPA = Invasive Pulmonary Aspergillosis, mNGS = metagenomic next generation sequencing, mTGS = metagenomic third generation sequencing, tNGS = targeted next generation sequencing.

A retrospective analysis of 296 patients with suspected pulmonary infection found that mNGS identified a broader range of pathogens and may be especially useful in immunocompromised patients.^137^ tNGS and mNGS were able to outperform conventional microbiological tests, however there was a signal towards greater discordance of tNGS and mNGS in the HIV positive patients.^138^

mNGS may have particular utility in certain patient and pathogen groups. For example, PJP diagnosis is notoriously difficult and typically replies on combination testing with BDG and pneumocystis PCR on induced sputum or BALF. Studies have demonstrated utility of metagenomics in diagnosing PJP in BALF of kidney transplant recipients.^139^

Another area where metagenomics has advantages over conventional testing is detection of mixed infections, which are more common in the profoundly immunocompromised. For example, in intensive care patients with severe community acquired pneumonia BALF mNGS was able to detect mixed infections better than conventional tests.^140^ It has also shown utility in diagnosis of mixed infection in renal grafts ^141^ and retrospective detection of rare and mixed infection in immunosuppressed patients with chronic kidney disease.^142^

Various sample types are being trialled. Plasma cfDNA has been studied in the USA using a metagenomic shotgun sequencing approach. The Karius platform was used on plasma in 106 patients with haematological malignancy, with a specificity of 96.6% but low sensitivity of 44.0% for proven/probable IFI. Use of BALF increased sensitivity to 72.2% but resulted in lower specificity of 83.3%. Notably, the results were not impacted by presence of mould-active prophylaxis.^143^

Others have also utilised plasma cfDNA: Xu et al. diagnosed *P. jiroveci* and *Mucorales* cases with mNGS where conventional mycological tests failed in patients with haematological disorders.^144^

In a 2025 study of 176 patients with fever of unknown origin, mNGS reduced time to diagnosis and in some patients avoided need for invasive sampling.^145^ This is being further investigated in a 2025 randomised controlled trial protocol which plans to prospectively compare plasma mNGS to conventional diagnostics in cancer patients presenting with febrile neutropenia.^146^

One issue common to sequencing technologies is how to disregard human nucleic material. Wesdorp *et al*. demonstrated proof of concept for their NGS pipeline which utilised an additional human reference genome for accurate diagnosis of *Aspergillus* infection in a small paediatric cohort.^147^

As an emerging technology, the best placement of mNGS for fungal infection is not yet determined and there are challenges limiting the expansion of use in a clinical setting. Its use is currently limited to large centres in high income countries, with need for send-away likely to increase TAT. Other issues include high costs, complex data interpretation, poor detection efficiency for thick wall fungi^148^ and contamination. Studies are underway to standardise read thresholds to differentiate infection from colonisation.^149^ With advancements in standardising procedures, interpretation criteria, improved nucleic acid extraction methods, and cost reductions, mNGS could become a routine diagnostic tool for fungal infections.^150^

### Biosensors, microfluidics, and lab-on-a-chip technology

Microfluidics, or lab-on-a-chip, technologies involve manipulating flow of fluids through channels in order to automate and miniaturise lab processes. They often utilise biosensor technology: the use of biological molecules to detect chemical reactions which are then transduced into detectable signals.^151^ Biosensors use diverse methods, including enzymes, nanoparticles, and ‘label-free’ techniques such as electrochemical impedance spectroscopy.^152^ The ideal lab-on-a-chip device would require minimal technical expertise, minimal reagent use and have all processes streamlined on one chip.

Microfluidic devices are already in widespread use within clinical infectious diseases, with products such as Cepheid GeneXpert now a standard for TB diagnosis, however, use in mycology lags behind. A 2024 review summarised the current progress in the field, pointing out the assays in development and the challenges encountered at each process on the chip.^153^

A number of commercially available multiplex assays utilise lab-on-a-chip technology to achieve their results. Early technologies such as the LightCycler® Sepsifast (Roche) had high resource requirements and have been phased out. Newer chips are now available such as the cobas® ePlex blood culture identification fungal pathogen (BCID-FP) panel and the BIOFIRE BCID2 Panel.^154,155^ These chips utilise PCR and microfluidic technology to provide microorganism ID from positive blood culture.^155^ The ePlex BCID-FP panel has been validated against a large number of clinical (141) and spiked (725) samples.^156^

For respiratory samples, there is the cobas® eplex respiratory pathogen panel to characterise lower respiratory tract infections.^157^ Others are in development: The MoiM Dx *Candida* 9-plex (MoiM) assay performed comparably to the BCID2 with a TAT of 90 min.^158^

Other microfluidic devices have shown promise in ability to quickly identify and speciate *Candida* with a limit of detection of 10^1^–10^2^ CFU/ml and short processing time.^159^ Others have further improved TAT by avoiding a DNA amplification step, allowing detection of *Candida albicans* from whole blood in 2 h following lysis.^160^

Benefits of these technologies include faster turnaround time, reduced reagent use, and possibility for portability. There is also potential to create very inexpensive devices. However, most devices have used positive blood culture as a starting point, retaining reliance on culture and focusing largely on yeasts.

Microfluidic technologies have already been developed for detection of antibacterial resistance^161^ and there is now research into antifungal resistance. Microfluidics have been used to determine susceptibility of *Candida* spp. to amphotericin B and fluconazole.^162^ A lab-on-chip for PCR and detection of azole-resistance mutations exists, however it requires prior culture and nucleic acid extraction steps.^163^

Loop mediated Isothermal Amplification (LAMP) technology is harnessed for many microfluidic chips. LAMP assays are already used by the agricultural industry to detect post-harvest fungal pathogens,^164^ including *Aspergillus* strains which produce harmful flavatoxins.^165^ The assays are highly sensitive, with LAMP on a microfluidic chip able to detect blight mould in tomato plants down to a limit of 1pg DNA/µl.^166^

In humans, there is a commercially available microfluidic LAMP *Candida auris* assay,^167^ with another assay for *Candida* identification (Eazyplex®Candida ID) also showing promising results.^168^ A LAMP lab-on-a-chip system has been described which identified Trichophyton DNA with a specificity of 100% and a detection threshold of 1 × 10^2^ copies/μl.^169^

Microfluidic devices can use smaller sample volumes than traditional testing, but the implications for this on sample characteristics are not well established.^170^ One problem that limits sensitivity of both traditional culture and of blood PCR is the low abundance of fungal pathogen compared to blood cells. A microfluidic protocol has been developed that can be used to purify *Candida* from blood samples, due to the size differential between yeast cells and human blood cells.^171^ Along this vein, authors have succeeding in using microfluidic cell washing to concentrate *Candida* cells, improving their lower limit of detection.^172^

To overcome the limitation of expensive production materials, microfluidic devices using paper and capillary action have been developed. These have the potential to become a portable, inexpensive, rapid, point-of-care diagnostic tool in resource-limited settings.^173,174^ Clinical application and commercialisation have been delayed due to challenges of uncertain market viability, a lack of standardisation and production problems.^175,176^

The next steps in lab-on-a-chip technology may be addition of nanofluidic components. Nanometric channels can be designed to help sort single molecules such as DNA and proteins.^177^ The properties of nanoparticles of elements such as gold and nanomaterials such as graphene are also being explored to assist with fungal diagnostics.^178^ Plasmonic nanoparticles which, simply put, are particularly sensitive to changes in the refractive index of their medium, can now be engineered for a variety of uses^179^ and nano-spectroscopy is being investigated for *Aspergillus* detection, though still in the experimental stage.^180^

Novel technologies such as giant magnetoresistance (GMR) also show applicability to the medical biosensor field. Exquisite sensitivity to changes in magnetic field enables the sensor to detect tiny levels of desired biomarkers.^181^ Authors Vergidis et al. tested a novel, GMR-enabled multiplex PCR to detect cfDNA of 18 common fungal species. Their proof-of-concept study demonstrates combination of barcoding with GMR to create a chip able to identify four yeasts.^182^ Further validating studies are required with higher numbers of patient samples.

For lab-on-a-chip devices to reach their potential, and for development to be clinically relevant to IFI patients, there is a need for greater collaboration between the engineering and medical scientific communities.^183^

**Figure 3:**
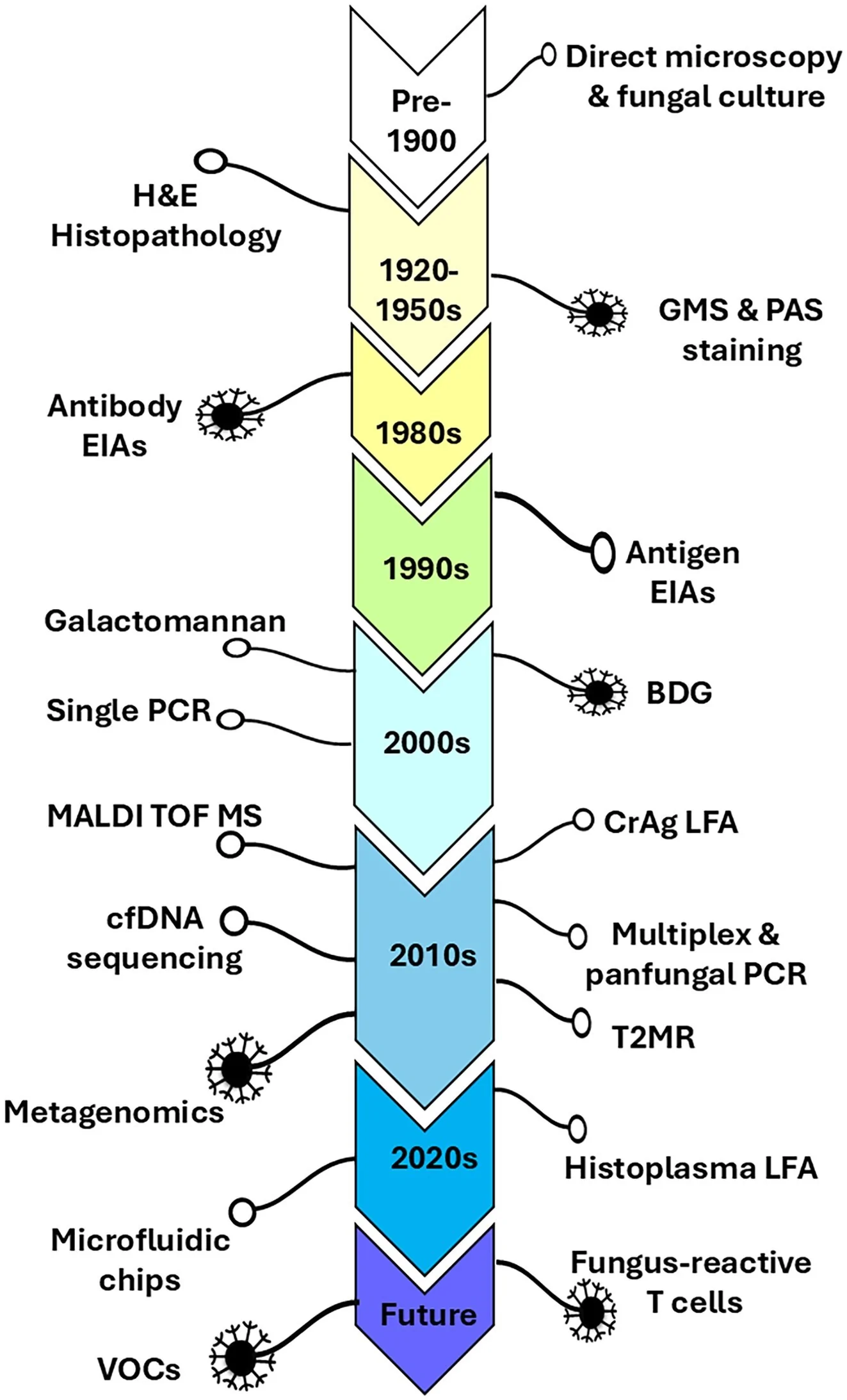
Timeline of key advancements in fungal diagnostics for clinical use. H&E hematoxylin and eosin stain; GMS Gomori Methenamine Silver; PAS Periodic Acid-Schiff; EIAs Enzyme Immunoassays; BDG 1,3-Beta-D-glucan (BDG) assay; PCR Polymerase chain reaction; MALDI TOF MS Matrix-Assisted Laser Desorption/Ionization Time-of-Flight Mass Spectrometry; cfDNA cell-free DNA; T2MR T2 Magnetic Resonance; CrAg cryptococcal antigen; LFA lateral flow assay; VOC volatile organic compounds ALT TEXT: This is a visualisation of the chronology of development of assay types for fungal diagnostics by decade from 1900s to 2020s.

Figure 3 places fungal diagnostics on a timeline moving from the past through to the future.

### Volatile organic compounds

Breath-based diagnostics represent a novel, non-invasive method to diagnose respiratory infections. VOCs are thought of as ‘unique chemical fingerprints’, released by fungi during quorum sensing and as a by-product of metabolism. The specific interaction between the fungus and the host during infection adds to this distinctiveness.^184^ Measuring fungal VOCs by gas chromatography and MS (GC-MS) provides a potential non-invasive diagnostic tool for respiratory fungal infections.^185,186^ Most studies at present are based *in vitro*, and are limited by the range of VOCs that can be detected with the aforementioned technology. Another form of MS: Thermal desorption-single photon ionisation-MS (TD-SPI-MS), has been trialled in the context of chronic pulmonary aspergillosis with some promising starting data.^187^ Furthermore, a pilot study is recruiting to analyse VOCs produced during invasive aspergillosis in lung transplant recipients using these spectrometry methods.^188^

However, despite proof-of-concept studies being published since 2013,^189^ this technology is not yet within clinical practice. Perhaps part of the delay is the uncertainty around which *in vitro* results are applicable in the human host: The majority of fungal volatilomes remain uncharacterised in human breath samples.^190^ Over the past decade, studies have attempted to compare data from VOCs isolated *in vitro*, to see if the same chemicals are released during human infection, with variable success. ^190–193^ Other fungi in addition *to Aspergillus* spp. have been studied including *Candida* spp., ^192^*Rhizopus* spp. and *Coccidioides* with limited data at present.^190^

Standardisation of collection and analysis methods is needed for this work to expand. Further studies are required, in-particular researching the endemic mycoses, where other diagnostics, such as serology, are of limited value, especially in immunosuppressed cohorts.

### Fungus-reactive T cells

Interrogation of fungus-reactive T cells has been described as a mode of diagnosing IFI. This is a non-invasive technique in which whole blood or peripheral blood mononuclear cell (PBMC) samples are stimulated with fungal lysates, then the number of reactive T cells is quantified with flow cytometry.^194^ The benefits include being a culture-independent technique and avoiding the risks of invasive sampling.

This technique has been used to diagnose mucormycosis and invasive aspergillosis in immunocompromised patients, including those on antifungal prophylaxis, with encouraging results. ^195,196^

While most studies focus on mould detection*, Candida*-reactive T cells also show promise. In a pilot study of 26 patients and 96 healthy donors, authors were able to classify invasive candidiasis patients with a sensitivity of (81.3%) and specificity of 100%. Drawbacks include the need for PBMC and that the sensitivity was limited by cell autofluorescence. ^194^

Isolation of PBMCs is time consuming and requires specialised laboratory staff. The use of whole blood is less well-described. A 2020 paper reports that by stimulating whole blood with additional co-factors they were able to achieve detection rates comparable or higher than with PBMCs.^197^

The interactions of the host adaptive immune system with fungal pathogens are a complex area, and it remains difficult to distinguish colonisation, allergy and invasive infection. ^198^ Differentiating environmental mould exposure and invasive disease is one area of ongoing research, as fungus-reactive T cells have been demonstrated among healthy adult PBMC and shown to correlate with environmental mould exposure, with trends of increasing positivity based on length of exposure and number of exposure risk factors. ^199^

*In vitro* application of immunosuppressive drugs, but not antifungal drugs, reduced the reactive T cell detection rates. ^200^ This remains to be investigated clinically but points to the problem that the patients at highest risk of IFI are likely to lack effective T cells, either due to underlying disease, or iatrogenic immunosuppression. This is demonstrated in a prospective cohort study of 115 at risk patients. While excellent sensitivity (100%) and specificity (81%) were achieved in analysed samples for diagnosis of IFI, a large number of samples were unable to be analysed due to low T cell numbers.^201^ The future applicability of fungus-reactive T-cell detection assays will depend on overcoming these hurdles.

Table 3 summarises new fungal diagnostics in development and their potential applications.

**Table 3:** Summary table of fungal diagnostics in development.

Diagnostic	Potential TAT	Benefits	Barriers
**Metagenomic NGS and TGS**	<10 h	Pathogen-agnostic, potential for non-invasive sampling	Expensive, complex interpretation
**cfDNA PCR**	<10 h	Non-invasive	Low cfDNA abundance, contamination risk
**Volatile organic compounds**	Minutes	Non-invasive, rapid, point-of-care	Unstandardised collection and analysis
**Novel mAbs**	Minutes (LFA) to hours (EIA)	Non-invasive. LFAs rapid.	Inter-species cross-reactivity
**Microfluidic chips**	Minutes to hours	Point-of-care, rapid, simple to use	Reliance on blood culture step. Low pathogen abundance
**Fungus-reactive T cells**	48 h+	Non-invasive; specific	Pre-analytic handing of PBMC requires specialised staff, expensive

TAT = turnaround time; NGS = next-generation sequencing; TGS = third-generation sequencing; cfDNA PCR = cell free DNA polymerase chain reaction; mAbs = monoclonal antibodies LFA = lateral flow assay; EIA = Enzyme Immunoassay.

### Artificial intelligence, enhanced imaging

Conventional radiological imaging is a cornerstone of fungal diagnosis and can be powerfully combined with laboratory tests. It is beyond the scope of this paper but has been addressed in recent reviews. ^202,203^

Artificial intelligence (AI) is transforming all areas of our world, but the impacts on fungal diagnosis are as yet unrealised. The task of amalgamating disparate and conflicting evidence including clinical and laboratory parameters to arrive at a fungal diagnosis is a challenge that could be suited to a machine learning (ML) model.^204,205^ ML is particularly suited to image analysis, and models have been successfully trained in identification of fungal species from microscopy images and histopathology, including directly identifying *Candida* spp. from blood culture gram stains, potentially removing the need for subculture.^206–208^

A 2026 systematic review has assessed the current evidence for AI-based diagnostic tools in diagnosing fungal infections (both invasive and non-invasive). Authors conclude that there is significant promise, but insufficient evidence of real-world application, with most studies utilising retrospective data and a lack of randomised controlled trials.^209^

In conclusion, the field of fungal diagnostics is evolving fast. The limitations of conventional mycological tests include poor sensitivity, specificity, inadequate TAT and requirement for experienced laboratory staff. There is a pressing need to overcome these, given the rapidly progressive, fatal nature of IFI. Accurate diagnostics could drive a culture change in antifungal prescribing, improve patient-specific treatment and reduce mortality by reducing time to effective treatment. Molecular techniques have improved the situation; however, they can be costly, rely on correct and adequate sample types, require standardisation, maintenance and are subject to contamination. Upcoming novel diagnostics provide hope and a springboard for further innovation. Lessons can be learnt from collaboration with scientists working outside the biomedical field. Clinical interpretation will remain paramount as new diagnostics become available, with results understood in the context of each patient.
